# Traumatic Stress Induces Prolonged Aggression Increase through Synaptic Potentiation in the Medial Amygdala Circuits

**DOI:** 10.1523/ENEURO.0147-20.2020

**Published:** 2020-07-23

**Authors:** Jacob Nordman, Xiaoyu Ma, Zheng Li

**Affiliations:** 1 Section on Synapse Development and Plasticity, National Institute of Mental Health, National Institutes of Health, Bethesda, MD 20892,; 2 National Institute of General Medical Sciences, National Institute of Health, Bethesda, MD 20892

**Keywords:** aggression, medial amygdala, PTSD, synaptic plasticity, traumatic stress

## Abstract

Traumatic stress can lead to heightened aggression which may be a symptom of psychiatric diseases such as PTSD and intermittent explosive disorder. The medial amygdala (MeA) is an evolutionarily conserved subnucleus of the amygdala that regulates attack behavior and behavioral responses to stressors. The precise contribution of the MeA in traumatic stress-induced aggression, however, requires further elucidation. In this study, we used foot shock to induce traumatic stress in mice and examine the mechanisms of prolonged aggression increase associated with it. Foot shock causes a prolonged increase in aggression that lasts at least one week. In vivo electrophysiological recordings revealed that foot shock induces potentiation of synapses formed between the MeA and the ventromedial hypothalamus (VmH) and bed nucleus of the stria terminalis (BNST). This synaptic potentiation lasts at least one week. Induction of synaptic depotentiation with low-frequency photostimulation (LFPS) immediately after foot shock suppresses the prolonged aggression increase without affecting non-aggressive social behavior, anxiety-like and depression-like behaviors, or fear learning. These results show that potentiation of the MeA-VmH and MeA-BNST circuits is essential for traumatic stress to cause a prolonged increase in aggression. These circuits may be potential targets for the development of therapeutic strategies to treat the aggression symptom associated with psychiatric diseases.

## Significance Statement

Heightened aggression can be a blight on society and a symptom of many psychiatric diseases. In this study we show that traumatic stress produces an enhancement of aggression that lasts at least one week through potentiation of synapses between the medial amygdala (MeA) and the ventromedial hypothalamus (VmH) and bed nucleus of the stria terminalis (BNST). Depotentiation of these pathways immediately after foot shock suppresses the increase in aggression, while having no effect on anxiety-like, depressive-like, or non-aggressive social behaviors. This study identifies the MeA-VmH and MeA-BNST circuits and synaptic potentiation as neural substrates for traumatic stress-induced prolonged aggression increase and potential therapeutic targets in treating the aggression symptom of psychiatric illnesses such as PTSD.

## Introduction

Aggression is an evolutionarily adaptive behavior to protect oneself from harm and acquire resources for survival (Nelson and Trainor, 2007). However, increased and abnormal forms of aggression can be harmful and may be symptomatic of psychiatric disorders, including PTSD, antisocial personality disorder, borderline personality disorder, and bipolar disorder (Miczek et al., 2004, 2013; Nelson, 2006; American Psychiatric Association, 2013; Dorfman et al., 2014). Aggression can be influenced by prior agonistic and stressful experience. For example, in rodents, stressful life experience such as social deprivation can instigate unprovoked attack behavior, and this effect is exacerbated by traumatic stress induced by foot shock (Veenema, 2009; Toth et al., 2011; Chang et al., 2015, 2018; Zelikowsky et al., 2018; Chang and Gean, 2019). In humans, traumatic stress may trigger acute stress disorder and PTSD, and individuals with PTSD may exhibit increased aggression that lasts for years after the initial stressor (Nelson and Trainor, 2007; McHugh et al., 2012; American Psychiatric Association, 2013; Smerin et al., 2016; Taft et al., 2017). The neural circuits involved in traumatic stress-induced, prolonged aggression increase are largely unclear.

The medial amygdala (MeA), a subnucleus of the amygdala, is a key structure involved in social behaviors including aggression and behavioral responses to stressors (Rodgers, 1977; Müller and Fendt, 2006; Nelson and Trainor, 2007; Takahashi et al., 2007; Hong et al., 2014; McCue et al., 2014). The MeA is activated by attack behavior and traumatic stress, and its overaction is associated with abnormal displays of aggression (Potegal et al., 1996a,b; Rosen et al., 1998; Haller et al., 2006; Márquez et al., 2013; Hong et al., 2014). Stimulation of the MeA increases the likelihood of a future attack and modulates such behavioral responses to threats as risk assessment and defensive behaviors (Potegal et al., 1996a; Miller et al., 2019). Injection of serotonin into the MeA suppresses foot shock-induced aggression (Rodgers, 1977). Inhibition of MeA activity interferes with the processing of social cues to associate environmental cues with threats, and lesioning of the MeA suppresses fear responses to predator odors and decreases avoidance of physical stressors (Müller and Fendt, 2006; Takahashi et al., 2007; McCue et al., 2014; Twining et al., 2017). Notably, in humans the MeA is a successful neurosurgical target in the treatment of intractable escalated aggression (Mpakopoulou et al., 2008).

Neurons in the MeA project to multiple brain regions that influence aggressive behavior including the ventromedial hypothalamus (VmH), bed nucleus of the stria terminalis (BNST), and lateral septum (LS; Gomez and Newman, 1992; Canteras et al., 1995; Coolen and Wood, 1998; Haller et al., 2006; Nelson and Trainor, 2007; Hashikawa et al., 2016). VmH activation is predictive of future attacks and optogenetic activation of the VmH promotes intermale aggression while optogenetic inhibition suppresses it (Kruk et al., 1983; Lammers et al., 1988; Lin et al., 2011; Yang et al., 2013; Falkner et al., 2014; Lee et al., 2014). The BNST is also activated by attack, and activation of the BNST modulates attack behavior in cats and increases social and attack behavior against conspecifics in mice (Masugi-Tokita et al., 2016). Hyperaggressive animals show low LS activity, inactivation or lesioning of LS promotes aggressivity, and optogenetic and electrical stimulation of LS suppresses it (Siegel and Skog, 1970; Albert and Wong, 1978; Ramirez et al., 1988; Goodson et al., 2005; Wong et al., 2016). All three areas are activated by stress, and the VmH and BNST have been shown to regulate aggressive responses to perceived threats (Silva et al., 2013; Anthony et al., 2014; Butler et al., 2015; Miller et al., 2019). The VmH in particular mediates the acute increase in aggression after foot shock (Chang and Gean, 2019). Based on these findings, it is intriguing to test whether these MeA target areas are involved in traumatic stress-induced prolonged aggression increase.

Here, using a combination of in vivo electrophysiology and optogenetics, we show that traumatic stress-induced by foot shock causes an increase in aggression and potentiation of synapses between the MeA and the VmH and BNST. These effects last for at least one week and can be abolished by synaptic depotentiation induced by low-frequency photostimulation (LFPS) of the MeA. These results indicate that traumatic stress drives prolonged increases in aggression through potentiation of select MeA pathways and suggest potential targets in the treatment of increased aggression associated with traumatic stress.

## Materials and Methods

### Animals

All animal protocols were approved by the Animal Care and Use Committee of the National Institute of Mental Health (ACUC). C57BL/6 male mice were purchased from Charles River. We housed mice under a 12 h light (9 P.M. to 9 A.M.)/12 h dark (9 A.M. to 9 P.M.) cycle with *ad libitum* access to water and food to be able to perform behavioral testing during the day, as this is the mice’s dark phase when they are most active (Koolhaas et al., 2013).

### Surgery

Ten-week-old mice were used for viral injection alone and seven-week old mice were used for viral injection followed by optrode implantation. Mice were anaesthetized with isofluorane (3% for induction and 1% for maintenance) and then placed onto a stereotaxic frame (David Kopf Instruments). Bilateral craniotomy was made and 500 nl virus was injected into the MeA (AP: –1.50 mm; ML: ±2.1 mm; DV: –5.15 mm) using a 5-μl gas-tight Hamilton syringe (33-gauge, beveled needle) at a rate of 0.1 μl/min. After injection, the needle was left in place for an additional 5 min and then slowly withdrawn. Immediately after viral injection, ferrule-terminated optical fibers (100 μm in diameter, ThorLabs) were placed 100 μm above the viral injection site at the MeA or at a midline position above the VmH (AP: +1.5 mm; ML: 0 mm; DV: −5.7 mm), BNST (AP: +0.14 mm; ML: 0 mm; DV: −3.8 mm), or LS (AP: +0.38 mm; ML: 0 mm; DV: −2.2 mm). Optical fibers were secured to the skull using Metabond (Parkell), stainless steel screws (PlasticsOne), and dental cement (DuraLay). Mice recovered for three weeks before MeA stimulation or six weeks before stimulation of MeA axons at the MeA projection sites. Four mice died after viral injection (1.92% of total). Materials can be found in Table 1.

**Table 1:** Key resources table

Resource type	Specific reagent or resource	Source orreference	Identifiers	Additionalinformation
Bacterial or viral strain	AAV9.CaMKIIa.hChR2 (E123A)-eYFP.WPRE.hGH	Addgene	Catalog #35506-AAV9	
Bacterial or viral strain	pRRlsin.eGFP	This paper		
Commercial assay or kit	Metabond	Parkell	Catalog #S380	
Commercial assay or kit	Dental cement	DuraLay	Catalog #602-7395	
Commercial assay or kit	Vectashield HardSet AntifadeMounting Mediumwith DAPI	VectorLaboratories	Catalog #H-1500	
Organism/strain	C57BL/6J	Charles River	Strain code: 556	
Software; algorithm	SigmaPlot	IBM	https://www.ibm.com	
Software; algorithm	TopScan/ObjectScan	CleverSystems	http://cleversysinc.com	
Software; algorithm	Video Freeze Software	Med Associates	https://www.med-associates.com/product/video-fear-conditioning/	
Software; algorithm	Labview	NationalInstruments	http://www.ni.com/en-us/shop/labview/labview-details.html	
Software; algorithm	MATLAB R2013a	MathWorks		

**Figure 1.**
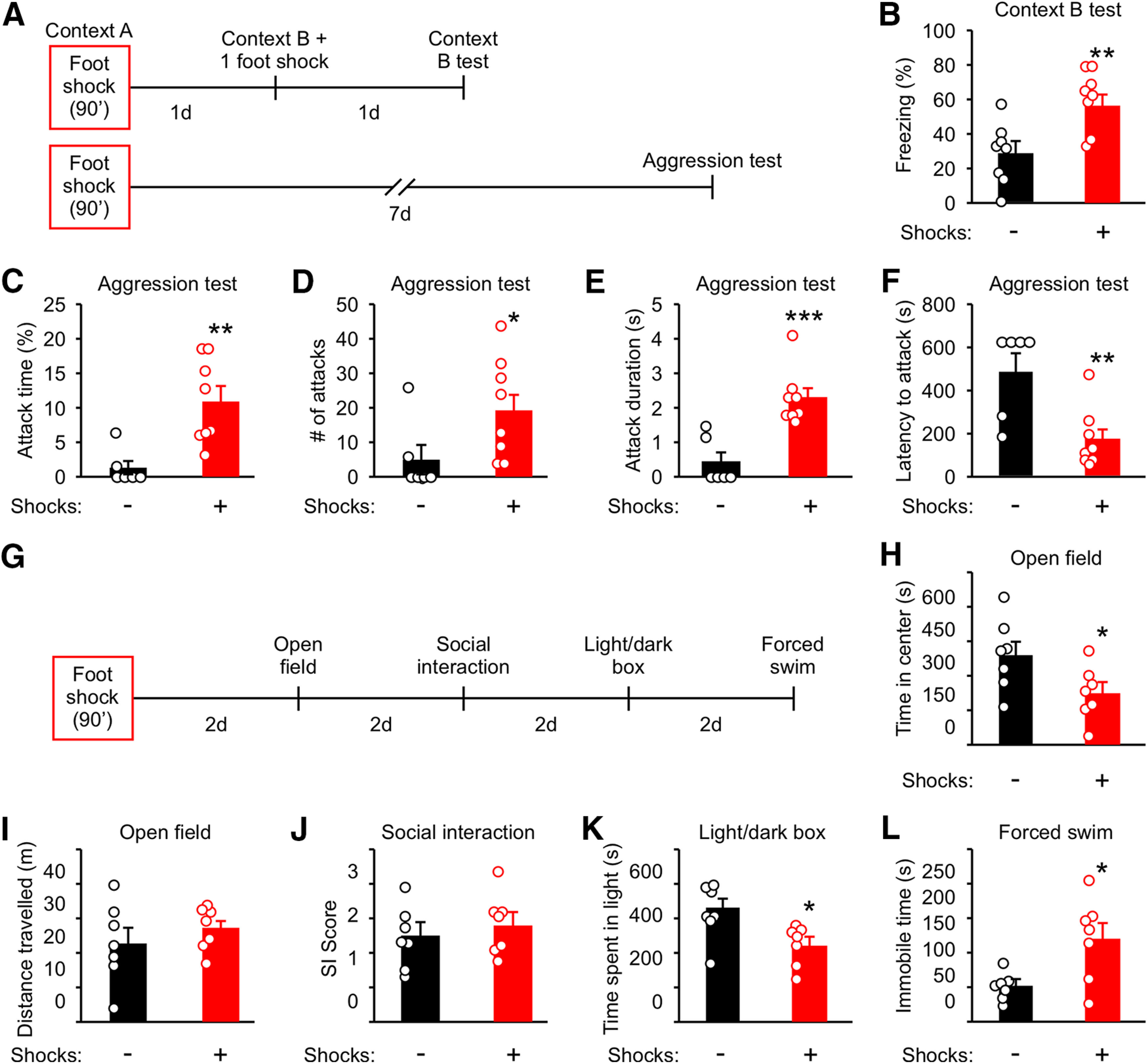
Traumatic stress enhances aggression, fear learning, and anxiety-like and depression-like behaviors while preserving non-aggressive social interactions. ***A***, Experimental schedule for ***B–F***. Ten-week-old mice were individually housed for three weeks before foot shock. Control animals were placed into Context A for the same amount of time but with no foot shocks applied. Different cohorts were used for ***B***, ***C–F***, ***H–L***. ***B***, Analysis of freezing behavior in Context B. ***C–F***, Analysis of attack behavior 7 d after foot shock. ***G***, Experimental schedule for ***H–L***. ***H***, Time in the center of the arena in the open field test. ***I***, Distance traveled during the open field test. ***J***, Ratio of time spent interacting with the cup containing a mouse to the time interacting with the empty cup (SI score) during the social interaction test. ***K***, Time spent in the light compartment during the light/dark box test. ***L***, Time spent immobile during the forced swim test. Data are presented as mean ± SEM; *p < 0.05, **p < 0.01, ***p < 0.001. Detailed statistics found in Table 2.

**Figure 2.**
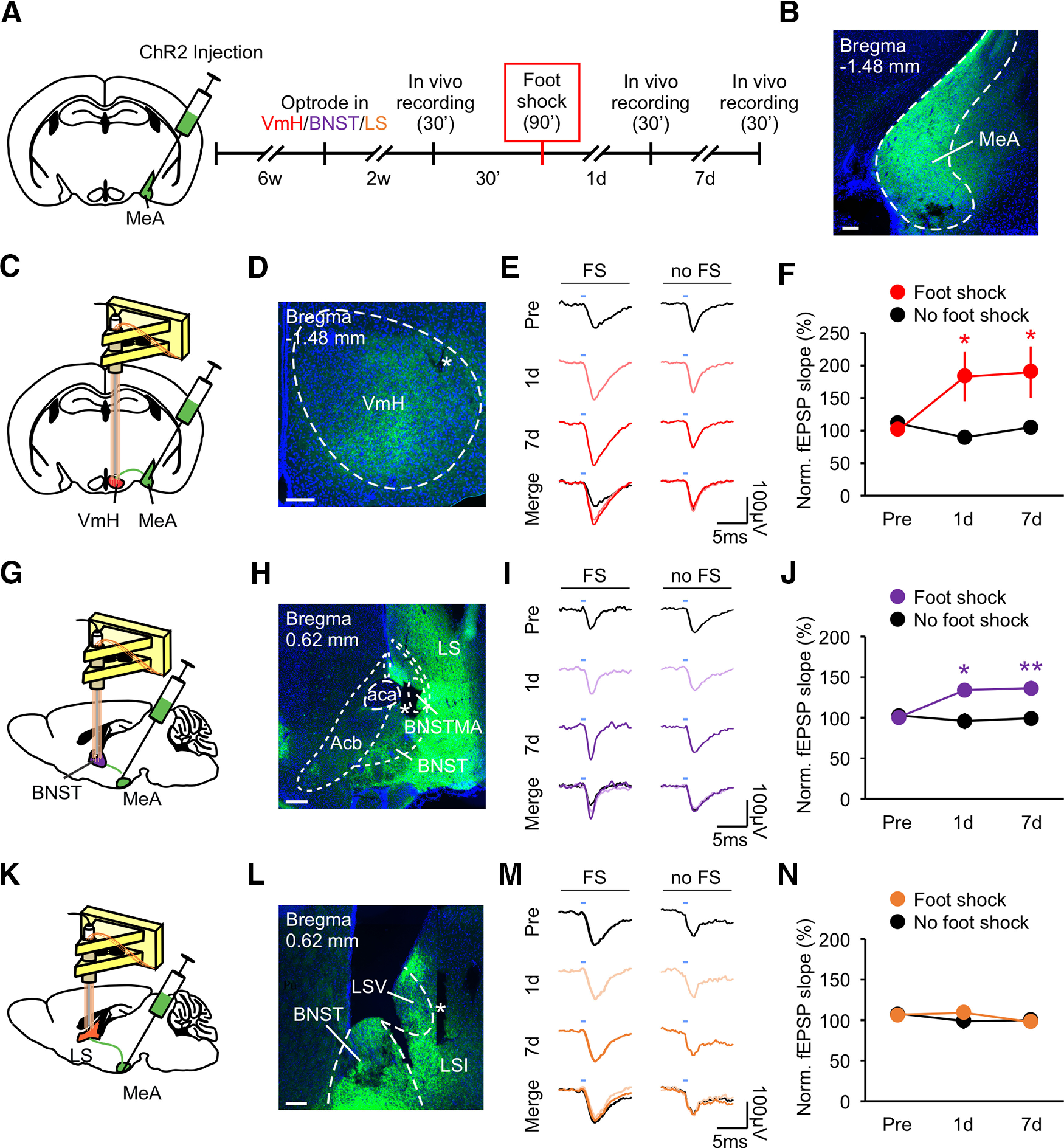
Traumatic stress induces LTP of MeA-VmH and MeA-BNST synapses. ***A***, Experimental schedule for *in vivo* electrophysiology. Individually housed mice were injected with ChR2 virus into the MeA and six weeks later were implanted with optrodes into the VmH, BNST, or LS. LFPs evoked by stimulating MeA axons at the VmH, BNST, or LS were recorded for 30 min. ***B***, Representative image of ChR2 expression at the MeA. Scale bar: 200 μm. ***C***, ***D***, ***G***, ***H***, ***K***, ***L***, Illustrations for the sites of viral injection and optrode placement (***C***, ***G***, ***K***) and representative images of ChR2 expression in MeA axons at the VmH (***D***), BNST (***H***), or LS (***L***). Scale bars: 200 μm (***D***), 200 μm (***H***), and 100 μm (***L***). ***E***, ***I***, ***M***, Representative traces of optically evoked responses at the VmH (***E***), BNST (***I***), or LS (***M***) recorded before, 1 d, and 7 d after foot shock or without foot shock. ***F***, ***J***, ***N***, Average slopes of 90 light-evoked fEPSPs recorded at the VmH (***F***), BNST (***J***), or LS (***N***) before (pre), 1 d, and 7 d after foot shock or without foot shock (normalized to preshock). Data are presented as mean ± SEM; *p < 0.05, **p < 0.01. See Extended Data Figure 2-1 for more details. Detailed statistics found in Table 2.

**Figure 3.**
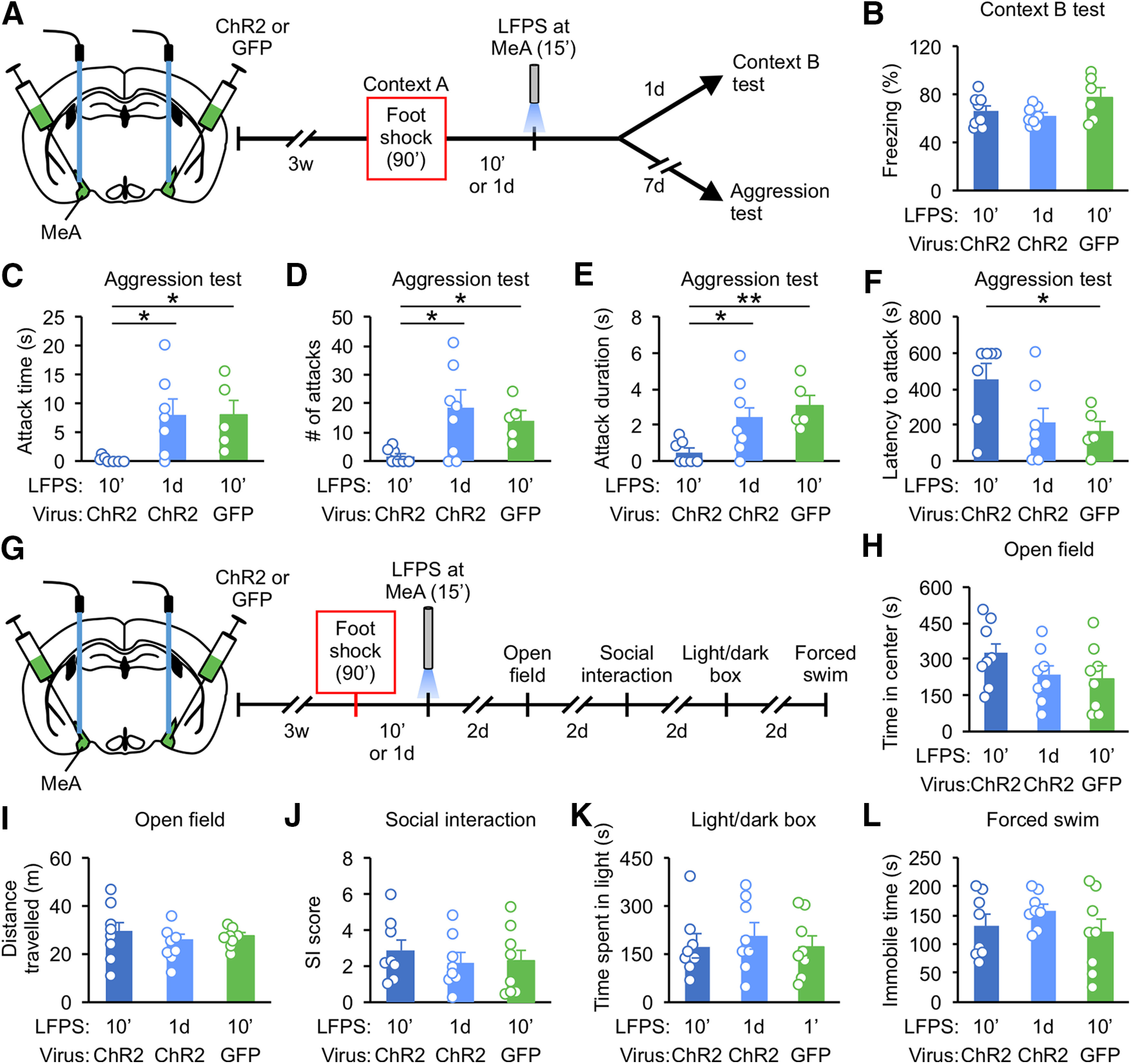
LFPS immediately after traumatic stress suppresses attack behavior. ***A***, Experimental protocol for ***B–F***. Mice were injected with ChR2 or GFP into the MeA and implanted with optical fibers above. After foot shock, LFPS was delivered immediately or 1 d after. Different mice were tested in ***B***, ***C–F***, ***H–L***. ***B***, Analysis of freezing behavior in Context B. ***C–F***, Analysis of attack behavior 7 d after foot shock. ***G***, Experimental protocol for ***H–L***. ***H***, Time in the center of the arena in the open field test. ***I***, Distance traveled during the open field test. ***J***, Ratio of time spent interacting with the cup containing a mouse to the time interacting with the empty cup (SI score) during the social interaction test. ***K***, Time spent in the light compartment during the light/dark box test. ***L***, Time spent immobile during the forced swim test. Data are presented as mean ± SEM; *p < 0.05, **p < 0.01. Detailed statistics found in Table 2.

**Figure 4.**
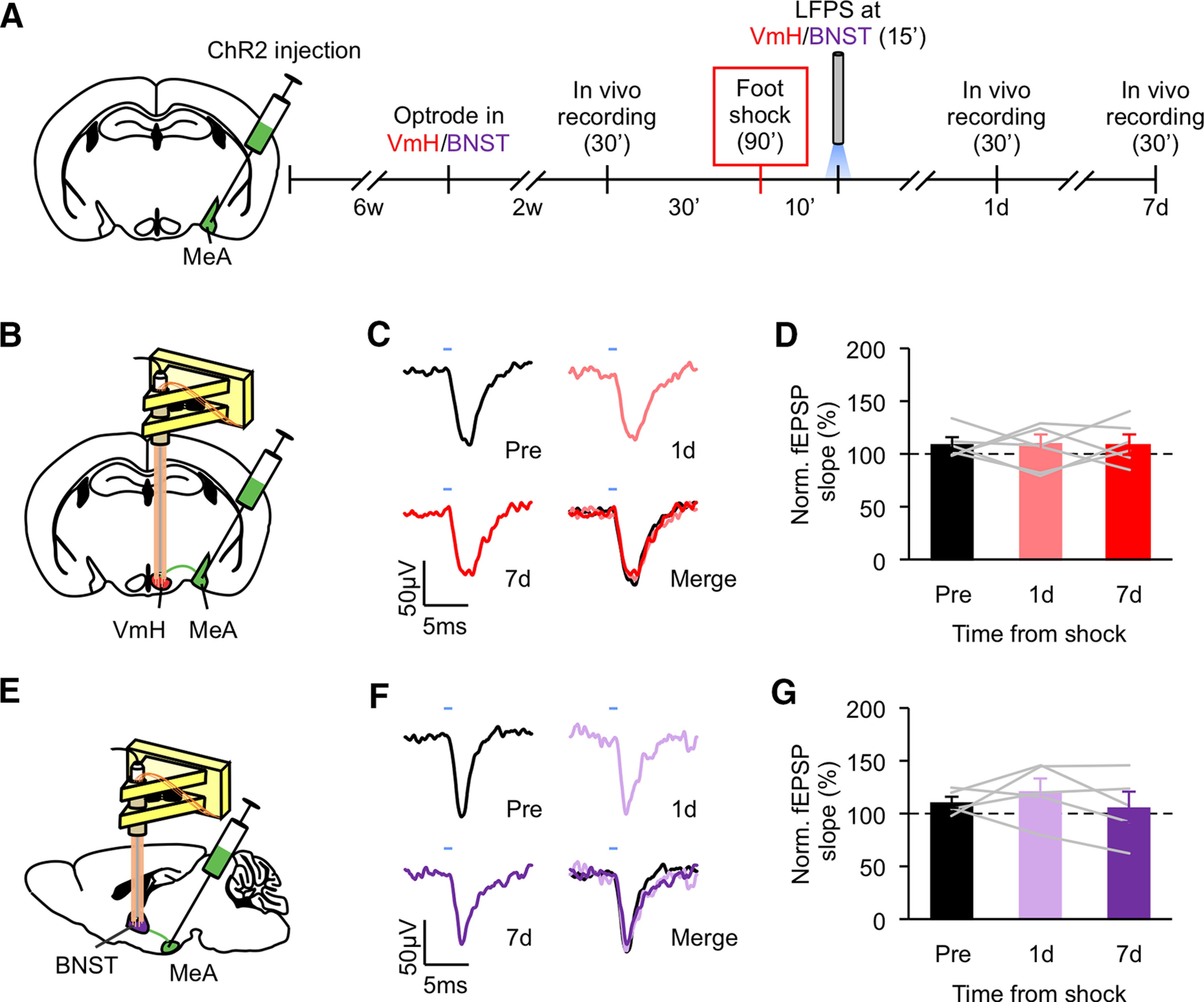
LFPS at the MeA-VmH and MeA-BNST synapses abolishes foot shock-induced synaptic potentiation. ***A***, Experimental schedule for *in vivo* electrophysiology. LFPS was delivered to the VmH and BNST immediately after foot shock. ***B***, ***E***, Illustrations for the sites of viral injection at the MeA and optrode placement at the VmH (***B***) or BNST (***E***). ***C***, ***F***, Representative traces of optically evoked responses at the VmH (***C***) or BNST (***F***) recorded before, 1 d, and 7 d after foot shock. ***D***, ***G***, Normalized slopes of light-evoked fEPSPs recorded at the VmH (***D***) or BNST (***G***) before and after foot shock. Data are presented as mean ± SEM. See Extended Data Figure 4-1 for more details. Detailed statistics found in Table 2.

**Figure 5.**
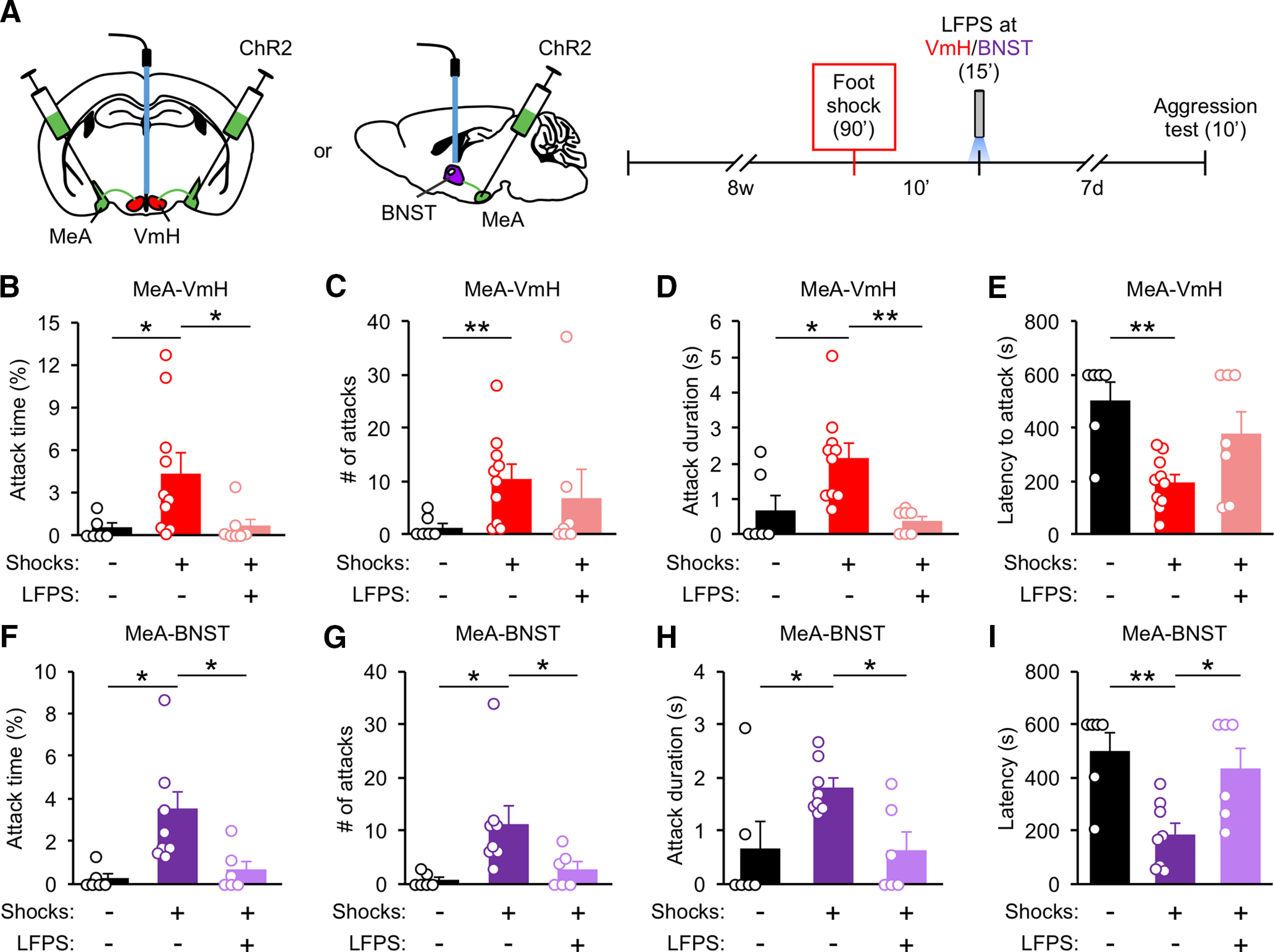
LFPS of the MeA-VmH and MeA-BNST synapses suppresses foot shock-induced aggression increase. ***A***, Experimental protocol. LFPS was delivered to the VmH or BNST immediately after foot shock. ***B–I***, Analysis of attack behavior 7 d after foot shock. Data are presented as mean ± SEM; *p < 0.05, **p < 0.01. Detailed statistics found in Table 2.

Key resources used in Figs. 1-5 and Extended Fig. 2-1 and Extended Fig. 4-1.

Some animals underwent a second surgery six weeks after viral injection to implant optrodes, which were used to record neural activities evoked by optical stimulation (Nabavi et al., 2014; Zhou et al., 2017). The optrode was inserted into the VmH, BNST, or LS at the same depth as the virus. The craniotomy was sealed with bone wax. Two stainless steel screws were inserted into the skull over the cerebellum and olfactory bulb to be used as ground and reference. Each optrode was outfitted with a microdrive and surrounded by a copper mesh to block external electrical noise. Two mice died after optrode implantation (0.96% of total).

### Behavioral tests and data analysis

All mice were individually housed after foot shock and surgery and for three weeks before baseline aggression testing. On the day of testing, mice were transferred to the behavioral room and allowed to acclimate for 1 h before commencing behavior experiments. Materials can be found in Table 1.

#### Foot shock and contextual fear memory test

Foot shock is a commonly used procedure to induce traumatic stress in rodents (Rau et al., 2005; Bali and Jaggi, 2015; Chang et al., 2015, 2018; Chang and Gean, 2019; Brivio et al., 2020). We adopted a foot shock protocol from Rau et al. (2005). On day 1, mice were placed into a fear conditioning chamber illuminated with white light (Context A) within a sound attenuating cubicle (Med Associates). After a 3-min exploration period, 15 electric shocks (0.4-mA, 1 s in duration) were administered through an electrified grate at random intervals of 240–480 s over the course of 90 min. Control mice did not receive foot shock.

For the contextual fear memory test, at 1 d after foot shock in Context A, mice were placed into the fear conditioning box modified with white plastic walls, no ambient light, and a background odor of 1% acetic acid (Context B). Mice were left to freely roam within the chamber for 192 s before a single 1 s, 0.4-mA shock was delivered via the electrified grate, and were removed from the chamber at 32 s after foot shock. On the following day, mice were placed into Context B for 8 min and 32 s and were monitored for freezing behavior. Freezing behavior was defined as an absence of all movement, excluding respiration, and was analyzed with Video Freeze software (Med Associates).

#### Aggression test

Mice were placed in a high-walled, novel cage and allowed to acclimate for 20 min before introduction of a younger, group-housed conspecific. Both mice were allowed to freely interact for 10 min. Animal behavior was captured with a video camera. Mice were tested for aggression before (baseline test) and after foot shock. Because a further increase in aggression may not be induced in mice with high aggression because of a ceiling effect (O'Donnell et al., 1981; Lee and Gammie, 2009, 2010), only mice exhibiting a total attack time of <3% in the baseline test, which was considered as low aggression in previous studies (Hong et al., 2014), were used for further analysis. Eleven mice (5.29% of total) were excluded based on this criterion. Videos of behavioral tests were reviewed and hand scored by a researcher blind to the experimental conditions. Aggressive behaviors such as chasing, boxing, pinning, and wrestling were identified as reported (Blanchard and Blanchard, 1977; Lin et al., 2011; Koolhaas et al., 2013; Hong et al., 2014; Golden et al., 2016).

#### Open field test

The open field test is a commonly used test for locomotion and anxiety-like behavior in mice (Crawley, 1999). Mice were transferred to the behavioral testing room at least 1 h before testing and then placed into a 49 × 49 cm open field arena for 30 min to freely roam. Distance traveled in the arena was analyzed using TopScan software (CleverSys).

#### Sociability test

The sociability test was modified from existing protocols (Silverman et al., 2010). During testing, mice were placed into a 49 × 49 cm arena with two inverted wire cups: one empty and the other containing an unfamiliar conspecific. Subject mice were allowed to freely investigate the arena for 30 min. All experiments were conducted under light with a luminescence level of 20 lux at the bottom of the arena (Kaidanovich-Beilin et al., 2011). Social interaction was analyzed using TopScan software (CleverSys) and scored as the ratio of time spent within 5 cm of the cup containing the animal over the empty cup.

#### Light/dark box

The light/dark box test is a commonly used test for anxiety-like behavior in mice (Hascoët and Bourin, 1998; Bourin and Hascoët, 2003). The test room was illuminated with 20 lux light. At time of testing, mice were placed in the light compartment of a light/dark box with dimensions of 46 × 27 × 30 cm, where one third of the box was dark and two thirds were transparent. Mice were then allowed to freely explore the test box for 11 min. The box was cleaned with 30% ethanol and water between runs. The test was recorded using a ceiling mounted camera and then analyzed using automated behavioral tracking software (TopScan/ObjectScan; CleverSystems).

#### Forced swim test

The forced swim test is a commonly used test for depression-like behavior (Porsolt et al., 1977; Can et al., 2012). Mice were placed into a large transparent plastic cylinder of height 30 cm and diameter of 20 cm, filled with fresh water to a height of 20 cm at 25°C. The test was conducted for 6 min and was recorded with a Digital camera. An index of despaired behavior was determined by duration of immobility as defined by no movement of limbs except for respiration. Mice were continuously monitored, and no mice drowned during the forced swim test.

### *In vivo* optogenetic stimulation

Optogenetic stimulation was performed via an optical fiber (ferrule fiber, ThorLabs) connected through a zirconia split sleeve and patch cord to a 473-nm laser (Coherent) under the control of an Optogenetics TTL Pulse Generator (Doric Lenses).

### Electrophysiology

#### Optrode fabrication

A microdrive was assembled from 3D-printed pieces, screws, and nuts, and then attached to a nano-miniature connector (Omnetics) with epoxy glue. The optrode was constructed with sixteen 30-μm diameter tungsten wires (California Fine Wire) surrounding a 100-μm diameter optical fiber. One end of the tungsten wire extended ∼300 μm beyond the tip of the optical fiber, and the other end was wired to pins of a nano-miniature connector. Impedance of each channel in the optrode was measured (usually ∼100 kΩ at 1 kHz) after construction.

#### *In vivo* electrophysiological recording in awake, freely moving mice

The nano-miniature connector on the microdrive was plugged into an amplifier (RHD2132, Intan Technologies). The amplifier was connected to an RHD2000 USB interface board (Intan Technologies) through a motorized commutator (Tucker Davis Technology). Electrical signals were filtered to obtain signals between 1 and 7500 Hz, sampled and digitized at 30 kHz by the amplifier, and recorded by RHD2000 Interface software (Intan Technologies). The 473-nm laser (Coherent) was controlled by a USB-6212 Bus-Powered DAQ Device (National Instruments) in Labview (National Instruments) virtual instruments. After a two-week recovery period, mice implanted with optrodes were placed into high-walled novel cages inside a grounded faraday cage and allowed to acclimate for 20 min before recording. Video recordings of animal behaviors were obtained via a ceiling mounted acA1300-200uc USB 3.0 camera (Basler) at 30 frames per second simultaneously with electrophysiological recording. Video and electrophysiological recordings were synchronized using Master-8 Pulse Stimulator (A.M.P.I.), which generated and sent an electrical signal for each light pulse and video frame to the RHD2000 recording software (Intan Technologies). Local field potentials (LFPs) were evoked by delivering 1–3 mW, 1-ms light pulses at 0.05-Hz through the implanted optical fiber. Laser power was adjusted to elicit field EPSPs (fEPSPs) with a clear early and late component. *In vivo* electrophysiological data were analyzed with custom-written MATLAB scripts. Only recordings with all baseline fEPSPs ≥ 90% of the average baseline fEPSP not significantly different from each other as determined by repeated measures ANOVA test, verified opsin expression, and correct targeting of optrodes were included in further analysis. Two mice (0.96% of total recorded mice) were removed based on these criteria.

### Production of GFP virus

HEK-293T cells were cultured on 15-cm plates coated with 0.1% gelatin in DMEM media supplemented with 10% fetal bovine serum (Thermo Fisher Scientific). When the cell reached 90% confluence, the medium was changed at 2 h before transfection. For transfection of each 15-cm plate, 10 μg pRRlsin.CMV.eGFP, 7.5 μg psPAX2, 2.5 μg pMD2G, and 1 μg pAdVantage packaging vector were added to 2 ml water containing 260 μl CaCl_2_ (2 m) and then mixed with 2 ml 2× HBSS (50 mm HEPES, 280 mm NaCl, and 1.5 mm Na_2_HPO_4_, pH 7.05). After incubation at room temperature for 2 min, the mixture was added to the culture plate dropwise. The medium was replaced with 15 ml UltraCULTURE medium (UltraCULTURE, 1 mm sodium pyruvate, 0.075% sodium bicarbonate, and 1× glutamine) at 16 h after transfection. The medium was removed 48 h after transfection and kept at 4°C. A total of 15 ml fresh UltraCULTURE medium was added to the plate and collected 72 h after transfection. The media collected at the two times were combined, filtered with 0.45-μm filter bottles, and centrifuged at 25,000 rpm for 90 min at 4°C (Beckman, SW28 rotor). The supernatant was removed, and the pellet containing the virus was dissolved by incubation with 100 μl 1× HBSS overnight at 4°C. For further purification of virus, the viral suspension was placed on the top of 1.5 ml 20% sucrose (in 1× HBSS) and centrifuged at 21,000 rpm for 2 h at 4°C (Beckman, SW55 rotor). The pellet was incubated with 100 μl 1× HBSS overnight at 4°C, aliquoted and stored at −80°C. The titer of purified virus was determined by transducing HEK-293T cells with a series of dilutions. All viruses used for *in vivo* injection had a titer of 10^9^–10^10^ IU/ml. We elected to use the GFP virus as it was routinely made and confirmed for transduction efficiency in our lab.

### Image acquisition and image analysis

Brain slices were imaged with a multi-slide fluorescent microscope (Zeiss Axio Scan) with a 10× (NA 0.45) objective to locate viral expression and optrode placement at regions of interest. The location of optrodes were determined from the electrode tract left in the brain tissue. In some images, boundaries can be seen between two adjacent areas because of uneven illumination between tiles. Z-stack confocal images were collapsed and analyzed with ImageJ by researchers blind to the experimental conditions.

### Statistical analysis

All data were presented as individual data points or expressed as mean ± SEM. SigmaPlot software was used for statistical analysis. To compare two groups, two-tailed Student’s *t* test was used if the data were normally distributed with equal variance, and Mann–Whitney *U* test was used if the data did not satisfy both the normality and equivariance tests. To compare three or more groups, one-way ANOVA or Kruskal–Wallis one-way ANOVA on ranks with Dunn’s methods and Tukey’s test for *post hoc* multiple comparisons were used. To analyze three or more groups injected with two different viruses, two-way ANOVA and Tukey’s test for *post hoc* multiple comparisons were used. To analyze *in vivo* electrophysiological data, repeated measures ANOVA was used for group differences across days, and two-tailed paired Student’s *t* test was used to compare day 1 and day 7 recordings to baseline; *p* < 0.05 was considered significant.

## Results

### Traumatic stress induces prolonged increases in aggression

Previous studies show that attack behavior increases at 30 min after traumatic stress induced by electric foot shock (Chang et al., 2015, 2018; Chang and Gean, 2019). To test whether the aggression increase following traumatic stress is long lasting, we administered a traumatic stress protocol by delivering 15-foot shocks at 0.4 mA randomly spaced over a 90-min period (Rau et al., 2005). Foot shocks were temporally randomized as unpredictable threat is more effective in producing sustained fear (Davis et al., 2010). Since only socially isolated mice show aggression increase after foot shock (Veenema, 2009; Toth et al., 2011; Chang et al., 2015, 2018; Zelikowsky et al., 2018; Chang and Gean, 2019), and heightened aggression is more prevalent in men than women suffering from PTSD and after social isolation in males than females in mice, *Drosophila*, and non-human primates (Gluck and Sackett, 1974; Rodgers and Cole, 1993; Tolin and Foa, 2006; Bubak et al., 2019), only male mice were used in this study.

Male mice (10-week-old) were individually housed for three weeks before shock. Control mice were left in the fear conditioning box for the same amount of time but with no foot shocks. One day after foot shock, mice were divided into two groups and tested for stress-enhanced fear learning (SEFL) and aggression (Fig. 1*A*). To test for SEFL, mice were placed into a novel environment (Context B) and received one-foot shock (1 mA) followed by assessment of freezing behavior in Context B 1 d after (Fig. 1*A*). Freezing in Context B was significantly enhanced compared with control mice (Fig. 1*B*), indicating that fear learning is enhanced by foot shock as previously reported (Rau et al., 2005). To evaluate the long-term effect of traumatic stress on aggression, aggression tests were performed 7 d after foot shock (Fig. 1*A*). Four parameters of attack behavior commonly used to examine aggression were measured: overall attack time, number of attacks, duration of each attack, and latency to the first attack (Hong et al., 2014; Golden et al., 2016; Todd et al., 2018). Consistent with previous reports (Haller et al., 2006; Nelson, 2006), foot shock significantly increased attack time, attack number, and attack duration, while decreasing latency to the first attack (Fig. 1*C–F*).

**Table 2:** Statistical table

Data	Method	Factor	*n*	*F*, *t*, or*U* DF	*F*, *t*, or*U* stat	*p* value	*Post hoc*test
Figure 1*B*	Mann–Whitney	Shocks	8, 8	*U* =	7.000	0.007	
Figure 1*C*	Mann–Whitney	Shocks	6, 8	*U* =	2.000	0.003	
Figure 1*D*	Mann–Whitney	Shocks	6, 8	*U* =	7.000	0.029	
Figure 1*E*	Mann–Whitney	Shocks	6, 8	*U* =	0.000	<0.001	
Figure 1*F*	Mann–Whitney	Shocks	6, 8	*U* =	4.000	0.008	
Figure 1*H*	Mann–Whitney	Shocks	7, 7	*U* =	8.000	0.038	
Figure 1*I*	Mann–Whitney	Shocks	7, 7	*U* =	18.000	0.456	
Figure 1*J*	Mann–Whitney	Shocks	7, 7	*U* =	16.000	0.534	
Figure 1*K*	Mann–Whitney	Shocks	7, 7	*U* =	5.000	0.011	
Figure 1*L*	Mann–Whitney	Shocks	7, 7	*U* =	7.000	0.026	
Figure 2*F*	Kruskal–Wallis one-way ANOVAon ranks with Dunn's methods	fEPSPs from shocks	6	*H*_(2)_ =	11.474	0.003	Tukey's
	One-way ANOVA	fEPSPs without foot shocks	3	*F*_(2,6)_ =	2.439	0.168	Tukey's
Figure 2*J*	One-way ANOVA	fEPSPs from shocks	5	*F*_(2,12)_ =	8.362	0.005	Tukey's
	One-way ANOVA	fEPSPs without foot shocks	4	*F*_(2,9)_ =	0.196	0.826	Tukey's
Figure 2*N*	One-way ANOVA	fEPSPs from shocks	4	*F*_(2,9)_ =	0.612	0.563	Tukey's
	One-way ANOVA	fEPSPs without foot shocks	3	*F*_(2,6)_ =	0.325	0.734	Tukey's
Figure 3*B*	Two-way ANOVA	LFPS from shocks	8, 8, 6	*F*_(1,19)_ =	2.548	0.127	Tukey's
	Two-way ANOVA	Virus	8, 8, 6	*F*_(1,19)_ =	0.383	0.543	Tukey's
Figure 3*C*	Two-way ANOVA	LFPS from shocks	7, 7, 5	*F*_(1,16)_ =	6.024	0.026	Tukey's
		Virus	7, 7, 5	*F*_(1,16)_ =	7.437	0.015	Tukey's
Figure 3*D*	Two-way ANOVA	LFPS from shocks	7, 7, 5	*F*_(1,16)_ =	4.595	0.048	Tukey's
		Virus	7, 7, 5	*F*_(1,16)_ =	10.286	0.005	Tukey's
Figure 3*E*	Two-way ANOVA	LFPS from shocks	7, 7, 5	*F*_(1,16)_ =	8.776	0.009	Tukey's
		Virus	7, 7, 5	*F*_(1,16)_ =	6.137	0.025	Tukey's
Figure 3*F*	Two-way ANOVA	LFPS from shocks	7, 7, 5	*F*_(1,16)_ =	5.844	0.028	Tukey's
		Virus	7, 7, 5	*F*_(1,16)_ =	4.969	0.04	Tukey's
Figure 3*H*	Two-way ANOVA	LFPS from shocks	7, 7, 5	*F*_(1,21)_ =	2.704	0.115	Tukey's
		Virus	7, 7, 5	*F*_(1,21)_ =	2.085	0.163	Tukey's
Figure 3*I*	Two-way ANOVA	LFPS from shocks	7, 7, 5	*F*_(1,21)_ =	0.223	0.641	Tukey's
		Virus	7, 7, 5	*F*_(1,21)_ =	1.603	0.219	Tukey's
Figure 3*J*	Two-way ANOVA	LFPS from shocks	8, 8, 8	*F*_(1,21)_ =	0.516	0.480	Tukey's
		Virus	8, 8, 8	*F*_(1,21)_ =	0.626	0.438	Tukey's
Figure 3*K*	Two-way ANOVA	LFPS from shocks	7, 7, 7	*F*_(1,21)_ =	0.000	0.984	Tukey's
		Virus	7, 7, 7	*F*_(1,21)_ =	0.364	0.553	Tukey's
Figure 3*L*	Two-way ANOVA	LFPS from shocks	8, 8, 8	*F*_(1,21)_ =	0.205	0.656	Tukey's
		Virus	8, 8, 8	*F*_(1,21)_ =	1.076	0.311	Tukey's
Figure 4*D*	One-way ANOVA	Time from shocks	6	*F*_(2,15)_ =	0.135	0.875	Tukey's
Figure 4*F*	One-way ANOVA	Time from shocks	5	*F*_(2,12)_ =	0.473	0.634	Tukey's
Figure 5*B*	Two-way ANOVA	Shocks	6, 10, 7	*F*_(1,20)_ =	0.018	0.895	Tukey's
		LFPS vs no stim	6, 10, 7	*F*_(1,20)_ =	5.588	0.028	Tukey's
Figure 5*C*	Two-way ANOVA	Shocks	6, 10, 7	*F*_(1,20)_ =	1.167	0.293	Tukey's
		LFPS vs no stim	6, 10, 7	*F*_(1,20)_ =	0.600	0.448	Tukey's
Figure 5*D*	Two-way ANOVA	Shocks	6, 10, 7	*F*_(1,20)_ =	0.292	0.595	Tukey's
		LFPS vs no stim	6, 10, 7	*F*_(1,20)_ =	12.715	0.002	Tukey's
Figure 5*E*	Two-way ANOVA	Shocks	6, 10, 7	*F*_(1,20)_ =	1.944	0.179	Tukey's
		LFPS vs no stim	6, 10, 7	*F*_(1,20)_ =	5.262	0.033	Tukey's
Figure 5*F*	Two-way ANOVA	Shocks	6, 8, 6	*F*_(1,17)_ =	0.155	0.698	Tukey's
		LFPS vs no stim	6, 8, 6	*F*_(1,17)_ =	7.25	0.015	Tukey's
Figure 5*G*	Two-way ANOVA	Shocks	6, 8, 6	*F*_(1,17)_ =	0.281	0.603	Tukey's
		LFPS vs no stim	6, 8, 6	*F*_(1,17)_ =	5.694	0.029	Tukey's
Figure 5*H*	Two-way ANOVA	Shocks	6, 8, 6	*F*_(1,17)_ =	0.007	0.937	Tukey's
		LFPS vs no stim	6, 8, 6	*F*_(1,17)_ =	6.973	0.017	Tukey's
Figure 5*I*	Two-way ANOVA	Shocks	6, 8, 6	*F*_(1,17)_ =	0.589	0.453	Tukey's
		LFPS vs no stim	6, 8, 6	*F*_(1,17)_ =	8.380	0.010	Tukey's
Extended DataFigure 4-1*B*	One-way ANOVA	fEPSPs from shocks	6	*F*_(2,15)_ =	6.752	0.008	Tukey's
Extended DataFigure 4-1*D*	One-way ANOVA	fEPSPs from shocks	5	*F*_(2,12)_ =	7.690	0.007	Tukey's

In addition to aggression, we examined the effect of foot shock on sociability, anxiety-like behavior, and depression-like behavior. Anxiety-like behavior was evaluated using the open field and light/dark box tests (Hascoët and Bourin, 1998), sociability was examined using the social interaction test (Silverman et al., 2010), and depression-like behavior was examined using the forced swim test (Porsolt et al., 1977). These tests were ordered as illustrated in Figure 1*G* and spaced with a 2-d interval to minimize the effect of stress related to behavioral testing. Foot shocked mice spent less time in the center of an open field arena (Fig. 1*H*), in the light compartment of a light/dark box (Fig. 1*K*), and were immobile for a longer duration during the forced swim test (Fig. 1*L*). No change was observed for distance traveled during the open field test (Fig. 1*I*) and in the social interaction score (SI, the ratio of time spent exploring a mouse to that exploring an object) during the SI test (Fig. 1*J*).

These results indicate that traumatic stress has long-term effects on aggression, anxiety-like behavior, and depression-like behavior and that the behavioral alterations of shocked mice are not caused by changes in locomotion or sociability.

### Traumatic stress induces long-term synaptic potentiation in MeA-VmH and MeA-BNST synapses

MeA and its downstream synaptic partners, the VmH, BNST, and LS, are key regions in the aggression circuit, and they have been implicated in traumatic stress-induced behavioral changes (Rodgers, 1977; Puciłowski et al., 1985; Shaikh et al., 1986; Nikulina et al., 2004; Nelson and Trainor, 2007; Silva et al., 2013; Butler et al., 2015; Masugi-Tokita et al., 2016; Wong et al., 2016; Hashikawa et al., 2017). The VmH in particular has been shown to mediate the acute effect of foot shock on attack behavior (Chang and Gean, 2019). Previously, it has been shown that 1-h restraint stress switches cannabinoid type-1 receptor-dependent synaptic plasticity in the BNST from long-term depression (LTD) to long-term potentiation (LTP) and that social instability stress causes changes in synaptic proteins at the MeA and LS (Glangetas et al., 2013; Hodges et al., 2019). Hence, it is possible that foot shock may alter synaptic transmission between the MeA and its downstream target regions. To test this possibility, we analyzed the strength of synapses between the MeA and the VmH, BNST, and LS in awake, behaving mice. To this end, six-week**-**old mice were injected with AAV channelrhodopsin-2 (ChR2) virus into the MeA and then implanted with optrodes into the VmH, BNST, or LS six weeks later (Fig. 2*A*,*C*,*G*,*K*). ChR2 expression was readily observed in MeA cell bodies (Fig. 2*B*) and MeA axons at all three projection sites by two weeks after virus injection when *in vivo* electrophysiological recordings were performed (Fig. 2*D*,*H*,*L*). Optically evoked fEPSPs were obtained at 30 min before, 1 d, and 7 d after foot shock, or without foot shock to rule out any potential handling effects. Optically evoked fEPSPs were analyzed by obtaining the slope derived by fitting the rising phase of the late component of the fEPSP (excluding the bottom and top 10%) with linear regression as described previously (Extended Data Fig. 2-1; Xiong et al., 2015; Zhou et al., 2017). Both the VmH and BNST showed sustained increases in fEPSPs after foot shock (Fig. 2*E*,*F*,*I*,*J*). Conversely, no changes were observed at the LS or in the no foot shock group (Fig. 2*E*,*F*,*I*,*J*,*M*,*N*). These results indicate that foot shock induces long-term synaptic potentiation in the MeA-VmH and MeA-BNST, but not in the MeA-LS, synapses.

10.1523/ENEURO.0147-20.2020.f2-1 Extended Data Figure 2-1 Methodology for analyzing light-evoked fEPSPs, related to Figure 2. ***A***, Raw traces of optically evoked fEPSPs (grey) and their average (red). ***B***, Averaged trace in ***A*** is labeled with early and late components of the fEPSP. ***C***, Magnification of the boxed area in ***B*** showing that slopes of fEPSPs are derived by fitting the rising phase (excluding the bottom and top 10%) of the late component of the fEPSP with linear regression. Download Figure 2-1, TIF file.

To assess whether synaptic potentiation in the MeA circuit mediates the increases in attack behavior from foot shock, we used an optogenetic synaptic depression protocol (900 pulses of 1-Hz stimulation, LFPS) which is proven effective *in vivo* (Nabavi et al., 2014; Zhou et al., 2017) to reduce synaptic strength. Ten-week-old socially isolated mice were injected with ChR2 virus or GFP virus into the MeA and optical fibers were implanted above. Three weeks later, mice were delivered 15 foot shocks and then returned to their home cages. In separate groups, mice were delivered LFPS immediately or 1 d after foot shock (Fig. 3*A*). Mice injected with ChR2 virus and stimulated with LFPS immediately, but not 1 d after, foot shock were less aggressive than mice injected with GFP virus and stimulated with LFPS (Fig. 3*C–F*). LFPS applied immediately or 1 d after foot shock had no effect on contextual fear memory, anxiety-like behavior, depression-like behavior, sociability, or locomotion (Fig. 3*B*,*H–L*).

These results indicate that foot shock induces potentiation of synapses between MeA and its synaptic partners and that there is a critical period to prevent foot shock-induced aggression increase via synaptic depression.

### Potentiation of the MeA-VmH and MeA-BNST synapses is required for foot shock-induced aggression increase

Having found that foot shock induces synaptic potentiation in the MeA-VmH and MeA-BNST synapses, we tested whether the effect of LFPS at the MeA is mediated by the MeA-VmH and MeA-BNST synapses. We injected ChR2 virus into the MeA of seven-week-old mice and implanted optrodes into the VmH or BNST six weeks later. Mice were then delivered foot shocks followed immediately after by LFPS (Fig. 4*A*). Optically evoked fEPSPs at MeA-VmH and MeA-BNST synapses were recorded *in vivo* before and after foot shock. fEPSPs recorded before, 1 d, and 7 d after foot shock were comparable (Fig. 4*B–G*). Importantly, we confirmed that LFPS depotentiated the MeA-VmH and MeA-BNST synapses that were potentiated by foot shock by recording fEPSPs immediately after foot shock and then immediately after LFPS (Extended Data Fig. 4-1).

10.1523/ENEURO.0147-20.2020.f4-1 Extended Data Figure 4-1 LFPS at the MeA-VmH and MeA-BNST synapses reverses foot shock-induced synaptic potentiation, related to Figure 4. ***A***, ***C***, Illustrations for the sites of viral injection at the MeA and optrode placement at the VmH (***A***) or BNST (***C***). ***B***, ***D***, Normalized slopes of light-evoked fEPSPs recorded at the VmH (***B***) or BNST (***D***) for 15 min before and 15 min after foot shock and then for 15 min immediately after LFPS. Each data point represents the average slope of the late component of nine evoked fEPSPs. Animal number is indicated in each panel in parentheses. Detailed statistics found in Table 1. Download Figure 4-1, TIF file.

To assess whether the MeA-VmH and MeA-BNST synapses are responsible for the effect of LFPS on foot shock-induced aggression increase, we applied LFPS to the MeA projections to the VmH and BNST immediately after foot shock and evaluated aggression 7 d later (Fig. 5*A*). Mice that received LFPS at the MeA-VmH and MeA-BNST projections immediately after foot shock displayed less overall attack time as well as shorter duration of each attack compared with mice that received foot shock but without LFPS (Fig. 5*B–I*). LFPS at MeA-BNST synapses also decreased the number of attacks and latency to the first attack (Fig. 5*C*,*E*).

Taken together, these findings indicate that potentiation of the MeA-VmH and MeA-BNST synapses underlie the prolonged aggression increase induced by traumatic stress through foot shock.

## Discussion

Traumatic stress can increase aggression (Nelson, 2006). Previous studies have shown that traumatic stress induced by foot shock can prime animals to attack and increase anxiety and depression-like behaviors (Chang et al., 2015, 2018; Chang and Gean, 2019). These effects were measured at 30 min after foot shock. The long-term effect of foot shock on attack behavior has not been explored. In this study, we find that foot shock causes an increase in attack that lasts for at least 7 d, while preserving locomotion and sociability. As the MeA is a key area mediating aggressive behavior, we further investigated the role of the MeA in this enhancement. *In vivo* electrophysiological recordings revealed that the MeA-VmH and MeA-BNST synapses were potentiated for up to 7 d after foot shock. No effects were found at the LS, another region involved in stress and aggression, and a downstream synaptic partner of the MeA. LFPS suppressed shock-induced potentiation of MeA-VmH and MeA-BNST synapses. LFPS also suppressed foot shock-induced aggression enhancement when applied to the MeA or the MeA-VmH and MeA-BNST synapses, indicating that potentiation of the MeA-VmH and MeA-BNST pathways is necessary for changes in attack behavior.

A natural question is how social exposure at 7 d after traumatic stress drives an aggressive response. One possibility is that potentiation of the MeA-VmH and MeA-BNST pathways results in a general increase in arousal that sets the mouse in a constant state of social alertness independent of social cues. High social arousal is expected to be reflected in social interactions. We see no changes in sociability or non-aggressive social behavior during the aggression test. Additionally, while LFPS of the MeA-VmH and MeA-BNST synapses suppressed foot shock-induced aggression increase, it did not alter non-aggressive social behaviors. These findings suggest that foot shock specifically affects aggressive behavior rather than general social interaction through the MeA-VmH and MeA-BNST pathways.

Previous studies have demonstrated a link between the VmH and BNST to attack behavior (Nelson and Trainor, 2007; Hashikawa et al., 2017). Both regions are also associated with stress responses. The VmH and BNST are activated by social and physical stress, with the VmH driving defensive responses to a predator and the VmH and BNST regulating anxiolytic responses to foot shock (Dennis, 1976; Jennings et al., 2013; Kim et al., 2013; Silva et al., 2013; Anthony et al., 2014; Butler et al., 2015; Zelikowsky et al., 2018). Recently, the VmH was shown to underlie attack behavior measured at 30 min after foot shock (Chang and Gean, 2019). The MeA is an afferent pathway for the VmH and the BNST, and these circuits have been implicated in aggression. MeA neurons expressing dopamine D1 receptors and projecting onto the VmH and BNST regulate fighting during the resident intruder assay (Miller et al., 2019). GABAergic neurons expressing the neuropeptide Y type 1 receptor (Npy1R) in the MeA receive input from VmH neurons and subsequently project onto the BNST to modulate territorial aggression during starvation (Padilla et al., 2016). It remains unclear, however, what role these pathways play in the aggression increase resulting from traumatic stress. We show here that the MeA controls foot shock-induced prolonged aggression increase through potentiation of the MeA-VmH and MeA-BNST synapses and that depotentiation of these synapses can suppress these increases in attack behavior. The MeA-VmH and MeA-BNST pathways may have non-redundant or overlapping functions in traumatic stress-induced aggression. A more detailed examination of these pathways is warranted to address this question.

It has been shown that foot shock induces attack behavior only after social isolation (Veenema, 2009; Toth et al., 2011; Chang et al., 2015, 2018; Zelikowsky et al., 2018; Chang and Gean, 2019) and that heightened aggression after social isolation is more prevalent in male than female animals (Gluck and Sackett, 1974; Rodgers and Cole, 1993; Bubak et al., 2019). In addition, high-aggression mice may not be able to further increase aggression because of a ceiling effect (O'Donnell et al., 1981; Lee and Gammie, 2009, 2010). We, therefore, elected to use socially isolated male mice that demonstrated low-aggression levels before foot shock to examine the effect of traumatic stress on aggression. It cannot be ruled out though that limiting our cohort to socially isolated, low-aggression mice may constrain the generalizability of our findings. It is possible that the increase in attack behavior after traumatic stress is attributable, at least in part, to sensitization of the MeA by social isolation. Thus, potentiation of the MeA circuit may be a mechanism for traumatic stress-induced aggression only under certain conditions. Future studies assessing the effect of social isolation on synaptic physiology of the MeA pathways and experiments using group-housed mice would address this question. However, optrode implantation poses technical challenges to postsurgery group housing.

Finally, the finding that LFPS can suppress foot shock-induced attack increase when applied immediately but not 1 d after shock indicates that depotentiation of MeA-VmH and MeA-BNST synapses is time constrained. This is consistent with the finding that there is a critical period after synaptic potentiation when depotentiation can be induced (e.g., 10 min in hippocampal slices; Chen et al., 2001; Huang and Hsu, 2001). The molecular mechanism for potentiation of the MeA-VmH and MeA-BNST synapses is an intriguing question for future studies. Neurotransmitter receptors including NMDARs, mGluRs, AMPARs, GABA-A receptors, and endocannabinoid receptors that are shown to mediate synaptic plasticity in other brain regions are potentially involved. Moreover, synaptic potentiation is often accompanied by the structural changes of synapses, such as formation and enlargement of dendritic spines, which support long-term information storage (Bolshakov et al., 1997; Toni et al., 1999; Bosch and Hayashi, 2012). Spine formation in the auditory cortex and at lateral amygdala-auditory cortex synapses has also been observed after foot shock stress (Yang et al., 2016; Lai et al., 2018). The effect of foot shock-induced aggression on the structure and formation of spines at MeA-VmH and MeA-BNST synapses is of interest to future studies.

## Synthesis

Reviewing Editor: Alfonso Araque, University of Minnesota Decisions are customarily a result of the Reviewing Editor and the peer reviewers coming together and discussing their recommendations until a consensus is reached. When revisions are invited, a fact-based synthesis statement explaining their decision and outlining what is needed to prepare a revision will be listed below. The following reviewer(s) agreed to reveal their identity: Christopher Cain, Po-Wu Gean.

Thanks for the time and effort to provide the excellent comments.

Based on your assessment, unless you indicate otherwise, I will submit the following summary statement:

The reviewers have found merit and interest on the findings reported. I concur with the reviewers’ comments expressing the interest of the study. However, they also expressed some concerns about several issues that need to be addressed and clarified. I also consider pertinent the concerns expressed.

Reviewers and I believe that the comments require less than two months of work.

I am looking forward to receiving a revised version of the manuscript with the reviewers´ comments addressed.

Alfonso Araque

Reviewing Editor

eNeuro

Comments of the reviewers:

Reviewer 1.

This is an interesting manuscript exploring the role of MeA and downstream targets (VmH, BNST, LS) in persistent escalation of aggressive behavior following trauma. Using multiple behavioral measures, pathway-specific optogenetics, and in vivo LFP recordings in mice they show that: 1) standard shock-trauma selectively enhances fear, anxiety, depression and aggressive behaviors for at least one week, 2) shock-trauma potentiates MeA transmission to VmH and BNST (but not LS), 3) this escalated aggression can be selectively blocked by a depotentiation protocol delivered to MeA or MeA axons in VmH or BNST immediately after trauma (but not later). The manuscript is well-written and the data are well-presented. The topic is important and ties nicely to other work employing the SEFL paradigm to model trauma-induced changes in learning and behavior relevant to psychiatric disorders like PTSD. There are a number of novel findings that are sure to advance our understanding of trauma-sensitive brain circuits that contribute to aggression. I have identified only 2 issues that may detract from the paper’s impact and will also list minor concerns/comments below.

Moderate concerns:

1. Consider removing the extinction experiments from the manuscript. They add little to the main story and are difficult to interpret. First, it is unclear why extinction to the trauma-shock context (Ctxt A) is done rather than extinction to the single shock context (Ctxt B). There is also no non-trauma control to demonstrate any SEFL-like extinction phenotype. The stats are reported in a strange way, but visually there seems to be significant extinction in the first experiment (Fig 1C), but not for controls in the depotentiation experiment (Fig 3C). Also, it’s unclear what a block of potentiation with LFPS should look like for extinction. The stats (1- and 2-way ANOVAs) present a confusing story. Overall these experiments distract from the other more interesting findings in the manuscript.

2. I have some concerns about the generalizability of the findings. The experiments were conducted only on males (no explanation for excluding females). Further, males failing to qualify as “low aggression” in initial screens were excluded (also without explanation). What percentage of males qualified as “low aggression”? Further, subjects were excluded if tissue damage occurred during the aggression tests. Further, subjects were excluded if greater than 30% of the aggression test was spent attacking (also no explanation for this). The authors also point out that trauma-induced escalation of aggression only occurs in single-housed mice. It seems important to very clearly report the rationale for exclusions and also the number of subjects excluded by each of these criteria in your experiments. I would also devote a section to the discussion about limitations of the findings given all these exclusion criteria.

Minor concerns:

1. please provide more details for the pre-screening test for aggression (lines 95-97). How was this conducted? What percentage of mice qualify as “low aggression”? Can you add a reference? What is the rationale for only studying low-aggression mice?

2. Line 113: make it clear here that mice underwent a second surgery.

3. The rationale for using a reverse-light cycle is never addressed. Is this necessary for the aggression effects reported here or elsewhere? Also, only some of the experiments explicitly state which phase of the light:dark schedule was used for the test.

4. line 138 notes that mice were also singly housed after the footshock session for aggression tests. Was this true for other behavioral assays too?

5. line 174: how often were tests terminated if mice couldn’t maintain floating behavior? Were all data for these mice also excluded?

6. lines 205 & 208 refer to Figure S6, but there were no supplemental figures included in my files for review. Maybe this refers to a previous version of the manuscript?

7. Lines 209-210: describe this baseline test in more detail. How long were the BL tests? How many fEPSPs were collected to determine whether or not to include the subject? How many subjects were excluded by this criterion?

8. Line 212: the rationale for creating your own GFP virus was never clear. Why not buy a similar virus but without the ChR2?

9. Kruskal-Wallis ANOVA on Ranks never mentioned in Methods stats section but used in Fig2F

Reviewer 2.

This is an interesting study examining the effects of acute stress (footshocks) on the MeA-BNST and the MeA-VMH synapses, which in turn promoted aggression in the socially isolated mice. The authors showed that electrical shocks induced aggression in social isolated mice concomitant with increased neuronal activity in the VMH and BNST. The ChR2-mediated low frequency photo-stimulation (LFPS) following footshocks reduced shock-induced aggression. Furthermore, immediately after electrical shocks, ChR2-mediated LFPS on the nerve terminals of the MeA neurons projecting to the VMH and BNST blocked shock-induced prolonged synaptic potentiation and reduced aggression on the post-shock 7th day. The authors conclude that potentiation of the MeA-VmH and MeA-BNST circuits is essential for the prolonged effect of stress on aggression.

The study is interesting. However, several issues need to be clarified, particularly the proper control experiments are required to strengthen the conclusion.

1. The authors claimed footshocks induces potentiation of the MeA-VMH and MeA-BNST synapses and the MeA activation controls shock-induced long-lasting aggression in social isolated mice. It raises a question: whether social isolation makes the MeA sensitive to footshocks or footshocks activates the MeA to induce long-lasting aggression. The experiments using group-housing mice as a control would make it clear.

2. In Figure 2, authors transduced AAV-ChR2 into the MeA and recorded “optically evoked” fEPSP in vivo in the VMH, BNST and the LS. Here, a control group of SI mice without footshocks would rule out the handling effect and confirm shock-induced synaptic potentiation in the MeA-VMH and the MeA-BNST.

3. In Figure 4, there is no direct evidence showing LFPS induces depotentiation. The authors should record fEPSPs before and after footshocks, and after LFPS.

4. Alternatively, c-fos expression can be used as a marker of neural activity in the VMH and BNST to confirm that LFPS effects in the VMH and the BNST.

5. The manuscript requires some language editing and grammar correction.
