# Supplementary figures and images for: Traumatic Stress Induces Prolonged Aggression Increase through Synaptic Potentiation in the Medial Amygdala Circuits
Source: eNeuro. 2020 Jul 23;7(4):ENEURO.0147-20.2020. doi: 10.1523/ENEURO.0147-20.2020 (PMC7385664; doi:10.1523/ENEURO.0147-20.2020)

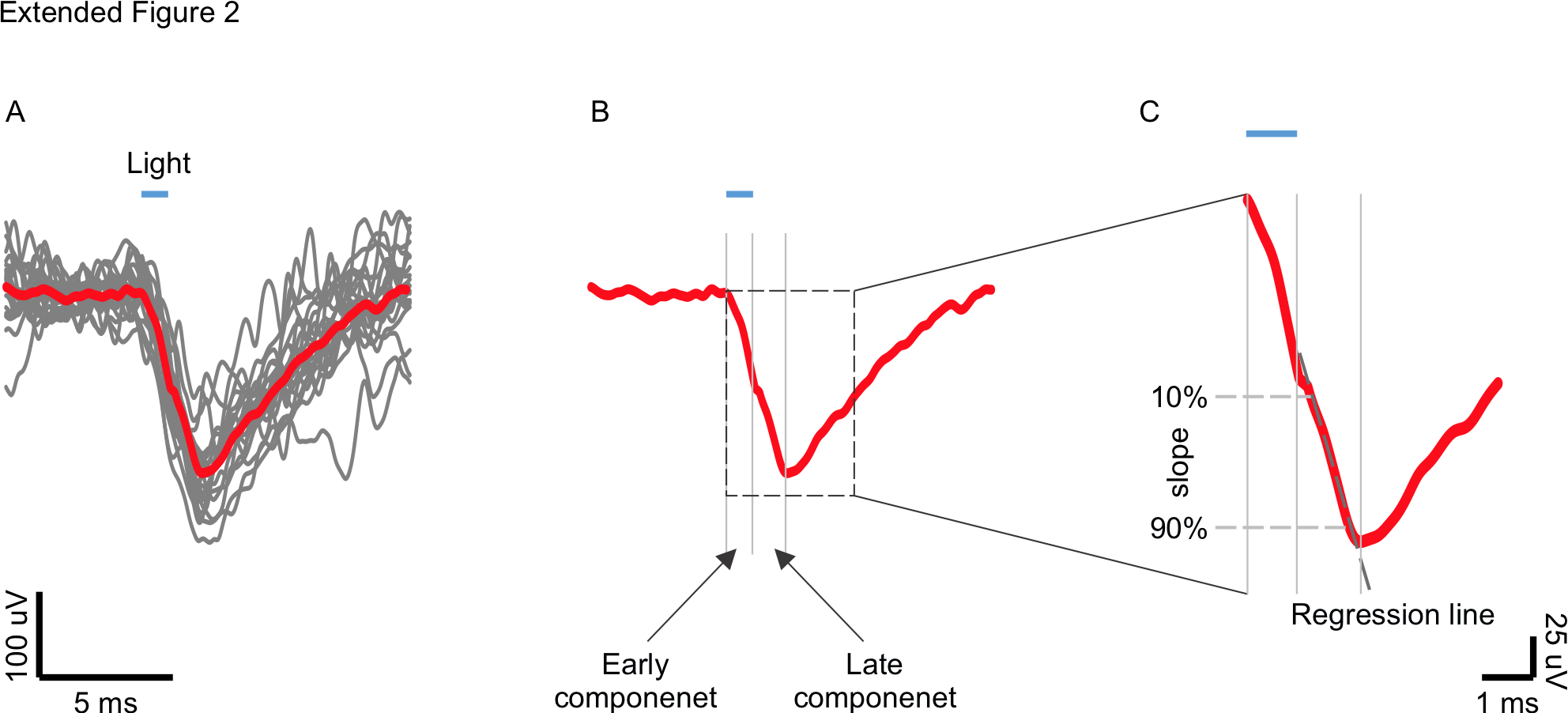

Supplement: Extended Data Figure 2-1 — Methodology for analyzing light-evoked fEPSPs, related to Figure 2. A, Raw traces of optically evoked fEPSPs (grey) and their average (red). B, Averaged trace in A is labeled with early and late components of the fEPSP. C, Magnification of the boxed area in B showing that slopes of fEPSPs are derived by fitting the rising phase (excluding the bottom and top 10%) of the late component of the fEPSP with linear regression. Download Figure 2-1, TIF file. [file enu-eN-NWR-0147-20-s01.tif]

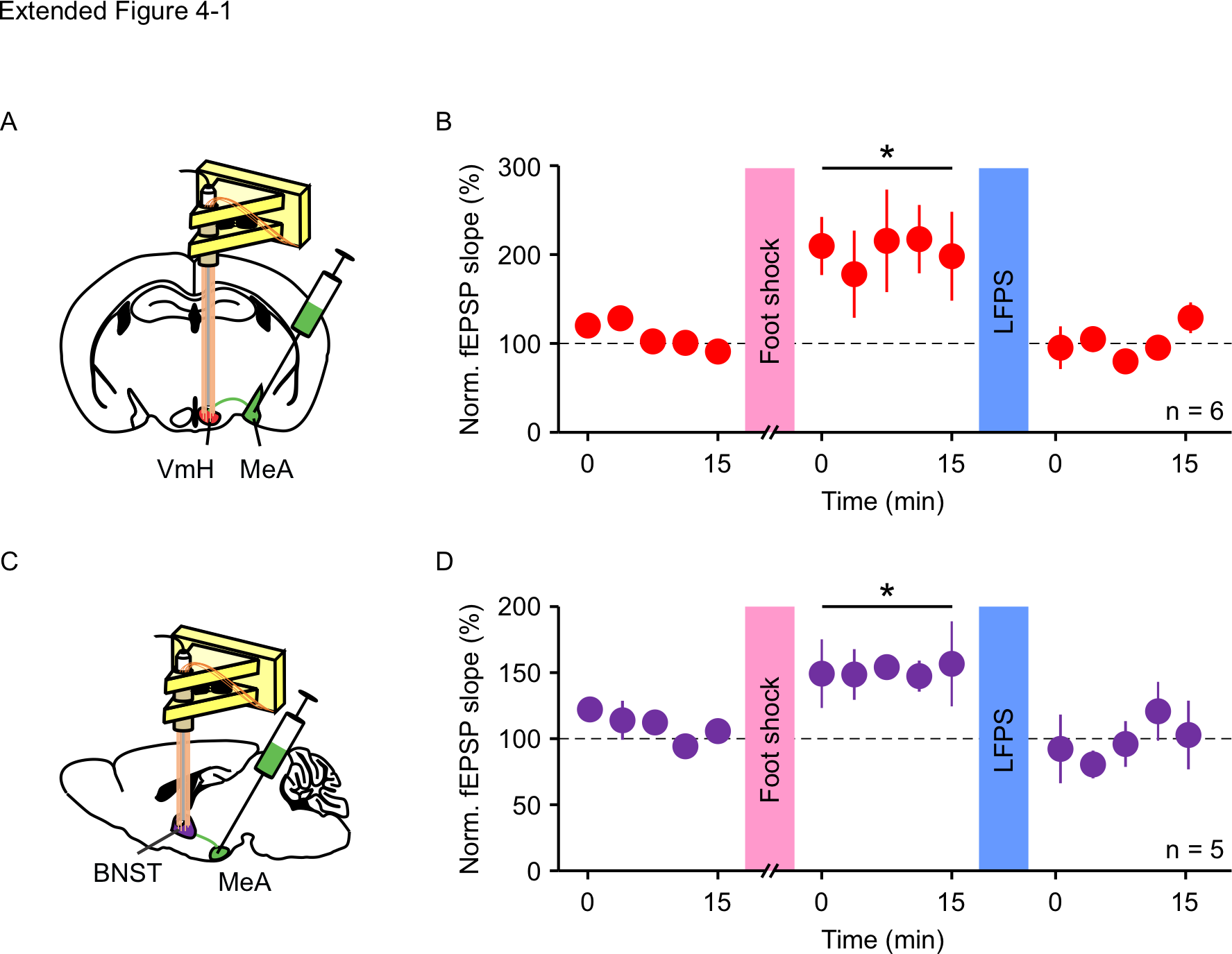

Supplement: Extended Data Figure 4-1 — LFPS at the MeA-VmH and MeA-BNST synapses reverses foot shock-induced synaptic potentiation, related to Figure 4. A, C, Illustrations for the sites of viral injection at the MeA and optrode placement at the VmH (A) or BNST (C). B, D, Normalized slopes of light-evoked fEPSPs recorded at the VmH (B) or BNST (D) for 15 min before and 15 min after foot shock and then for 15 min immediately after LFPS. Each data point represents the average slope of the late component of nine evoked fEPSPs. Animal number is indicated in each panel in parentheses. Detailed statistics found in Table 1. Download Figure 4-1, TIF file. [file enu-eN-NWR-0147-20-s02.tif]
